# Regulatory T and B cells in pediatric Henoch–Schönlein purpura: friends or foes?

**DOI:** 10.1186/s13075-024-03278-w

**Published:** 2024-02-16

**Authors:** Anne Filleron, Renaud Cezar, Marc Fila, Nastassja Protsenko, Kathleen Van Den Hende, Eric Jeziorski, Bob Occean, Thierry Chevallier, Pierre Corbeau, Tu Anh Tran

**Affiliations:** 1 IRMB, Montpellier University, INSERM U1183, Montpellier, France; 2 Department of Pediatrics, Nîmes University Hospital, Montpellier University, Service de Pédiatrie, Place du Pr R. Debré, 30029 Nîmes Cedex 9, France; 3 Department of Immunology, Nîmes University Hospital, Montpellier University, Nîmes, France; 4 Department of Pediatric Nephrology, Montpellier University Hospital, Montpellier University, Montpellier, France; 5 Department of Pediatric Infectious Diseases, Montpellier University Hospital, Univ Montpellier, INSERM, EFS, Univ Antilles, Montpellier, France; 6 Department of Epidemiology, Medical Statistics and Public Health, Nîmes University Hospital, Montpellier University, Nîmes, France; 7 UMR 1302 Desbrest Institute of Epidemiology and Public Health, INSERM, University of Montpellier, Montpellier, France; 8 Institute of Human Genetics, CNRS UMR9002, Montpellier University, Montpellier, France

**Keywords:** IgA vasculitis, T helper 3 cell, Regulatory T cells, Regulatory B cells, Kidney disease, Cytokines

## Abstract

**Background and objectives:**

Henoch–Schönlein purpura (HSP) is the most common immunoglobulin A-mediated systemic vasculitis in childhood. We studied immune dysregulation in HSP by analyzing regulatory T (Treg), T helper 3 (Th3), and regulatory B cell (Breg) subpopulations that might intervene in immune activation, IgA production, and HSP clinical manifestations.

**Methods:**

This prospective study included 3 groups of children: 30 HSP on acute phase, 30 HSP on remission, and 40 healthy controls (HCs) matched on age. Treg, Breg, and Th3 were analyzed by flow cytometry. Serum immunoglobulin and cytokine levels were quantified by ELISA and Luminex.

**Results:**

Treg frequencies were higher in acute HSP than in remitting HSP and HCs (6.53% [4.24; 9.21] vs. 4.33% [3.6; 5.66], *p* = 0.002, and vs. 4.45% [3.01; 6.6], *p* = 0.003, respectively). Activated Th3 cells (FoxP3 + Th3 cells) tend to be more abundant in HSP than in HCs (78.43% [50.62; 80.84] vs. 43.30% [40.20; 49.32], *p* = 0.135). Serum IgA, IL-17, and latency-associated peptide (a marker of the anti-inflammatory cytokine TGF-beta production) were significantly and inflammatory cytokines TNF-alpha, IL-1-beta, and IL-6 were non-significantly higher in HSP than HCs. Bregs were identical between the groups, but, in patients with renal impairment, Breg percentage was lower compared to those without. Treg removal in PBMC culture resulted in an increase in IgA production in HSP proving a negative regulatory role of Tregs on IgA production.

**Conclusions:**

In pediatric HSP, immune activation persists in spite of an increase in Th3 and Tregs. Th3 could be involved in IgA hyperproduction, inefficiently downregulated by Tregs. Lack of Bregs appears linked to renal impairment.

**Supplementary Information:**

The online version contains supplementary material available at 10.1186/s13075-024-03278-w.

## Introduction

Henoch–Schönlein purpura (HSP) or immunoglobulin A (IgA) vasculitis is the most frequent systemic vasculitis in children mainly aged 4 to 8 years [1, 2] mostly affecting boys (sex ratio 1.2–1.6/1). The annual incidence varies from country to country, 13 to 20/100,000 children [3–5]. HSP is characterized by a triad of purpura, arthritis or arthralgia, and abdominal pain. Diagnostic criteria were published in 2010 [6, 7]. The prognosis for HSP depends on the presence of renal involvement, occurring in 40% of children [8]. Two to 5% of children with HSP nephritis progress towards renal or end-stage renal failure.

HSP is a non-granulomatous IgA vasculitis of the small blood vessels. Skin biopsy reveals leukocytoclastic vasculitis with fibrinoid necrosis and an inflammatory perivascular infiltrate of neutrophilic polymorphonuclear and mononuclear cells. IgA deposits, the complement fraction C3, and fibrin are observed on the injured vessel walls. Kidney involvement varies from focal or diffuse mesangial proliferation to crescent deposits, with diffuse IgA deposits. Most studies on the pathophysiology of HSP have focused on the dysregulation of IgA production [9]: increased serum IgA levels, IgA-containing immune complexes which alter the vessels, presence of abnormal IgA1 glycosylation in renal disease, abnormalities in the regulation of clearance of IgA from the liver, and coagulation disorders (high d-dimer concentrations) [10–12].

Many contributing infectious triggering factors have been studied—mainly group A streptococcus infection but also other bacteria and viruses, exposure to drugs or toxic agents, and predisposing genetic factors (human leukocyte antigen [HLA] A2, A11, B35, mutation of the *MEFV* gene or heterozygous deficit in complement C) [13–15].

Regulatory T lymphocytes (Tregs) are a subgroup of helper CD4 + T lymphocytes (5–10% blood CD4 + T lymphocytes) able to downregulate immune activation. Tregs inhibit effector T and B cells, natural killer cells (NK), NKT lymphocytes, and antigen-presenting cells. Tregs act by cell-to-cell contact or by secreting anti-inflammatory cytokines such as IL-10 and transforming growth factor-β (TGF-beta). In mice, suppressing Tregs or impairing their function leads to severe autoimmune and inflammatory syndromes with multi-systemic involvement, like IPEX syndrome in humans [16].

In addition to Tregs, it has been shown that other T cells can prevent autoimmunity in rodents. Most of these cells, such as TR1 cells secreting IL-10, helper T3 (Th3) cells secreting TGF-beta, and certain CD4 − CD8 − and CD8 + CD28 − T cells, are adaptive regulatory cells: they acquire regulatory functions according to specific antigenic stimuli and cytokine environments.

TGF-beta induces IgA production by B cells stimulated with lipopolysaccharide (LPS) [17–19]. Thus, TGF-beta can act as a true isotypic switching factor for IgA production during the HSP acute stage [20].

Finally, a subset of B cells, regulatory B cells (Bregs) expressing IL-10, suppress CD4 + T cell-mediated production of pro-inflammatory cytokines [21–23]. In adult HSP nephritis patients, the proportion of Bregs was significantly lower than in healthy controls [24]. In HSP children, the Breg percentage and their ability to produce IL-10 were lower in patients with renal involvement and lowest in those with massive proteinuria [25].

The complete pathogenesis of HSP remains unknown. Certain T lymphocyte populations, including Treg and Th3, can modulate the IgA switching factor TGF-beta production. This suggests the possible role of natural and adaptive Tregs in the pathophysiology of HSP. The primary objective of our study was to analyze immune regulation in HSP.

## Patients and methods

### Patients

This prospective study included three groups of subjects: A—HSP on acute phase according to the EULAR/PRES/PRINTO 2010 classification; B—remitting HSP; C—healthy controls (HCs) matched on age with HSP patients. Subjects aged 3 to 18 years old were included from February 2015 to July 2017 in Montpellier and Nîmes university hospitals.

The exclusion criteria were patients with other autoimmune or inflammatory diseases and on immunosuppressive treatment, biologics, or antibiotics over the previous 3 months.

Demographic data and HSP involvement, biological parameters, and treatments were recorded. We analyzed the regulatory immune cells and soluble factors in HSP patients with biopsy-proven nephritis (HSPNb) or nephritis with urine test diagnosis (HSPNu) or without renal involvement (HSPw).

### Cell count of Tregs and other blood cell populations

From each patient, at inclusion, 10 ml of blood was collected in sodium heparin-containing vacutainer tubes.

Lymphocyte (CD3 + , CD4 + , CD8 + T, and CD19 + B lymphocytes, CD3-CD56 + NK cell) counts and percentages were established from fresh peripheral blood using CYTO-STAT tetraCHROME kits with Flow-Count fluorescent beads as the internal standard and tetra CXP software.

For T cell activation and B cell analysis, monoclonal antibodies conjugated with fluorescein isothiocyanate (FITC), phycoerythrin (PE), energy-coupled dye (ECD), PE-Cyanine5.5 (PC5.5), PE-Cyanine7 (PC7), allophycocyanine (APC), APC/Alexa700, or APC/Alexa750 (Beckman Coulter) were used in the following combinations: anti-human-CD45RA-FITC/anti-human-CD69-PE/anti-human-HLADR-ECD/anti-human-CD25-PC5.5/anti-human-CD197-PC7/anti-human-CD8-APC/anti-human-CD4-APC700/anti-human-CD3-APC750/anti-human-IgA-VioBright-FITC (Miltenyi Biotec)/anti-human-CD27-PC7/anti-human-CD19-APCA700. Blood was stained with a cocktail of antibodies and fixed with an IntraPrep Permeabilization Reagent Kit (Beckman Coulter).

For Treg analysis, direct immunostaining was performed on 50 μl of blood using the PerFix-nc kit (Beckman Coulter).

### Th3 lymphocytes

Blood samples were separated on Ficoll-Hypaque gradients to obtain peripheral blood mononuclear cells (PBMCs) stored in liquid nitrogen.

3 × 10^5^ cells were stimulated with a T Cell Activation/Expansion kit (Miltenyi Biotech). The cells were expanded in complete media (RPMI 1640 with 10% fetal calf serum, 50 µg/ml penicillin–streptomycin, and 2 mM l-glutamine) and incubated at 37 °C with 5% CO_2_ for 3 days. For the last 4 h, cells were stimulated with Phorbol 12-Meristate 13-acetate at 50 ng/ml (PMA), ionomycin at 1 µg/ml (Sigma Aldrich), and Brefeldin A 1X.

After stimulation, cells were stained using the PerFix-nc kit. Staining was performed with anti-human-CD25-PC5.5/anti-human-CD127-PC7/anti-human-FOXP3 − AF647/anti-human-CD4-APCA700/anti-human-CD3-APCA750 (Beckman Coulter) and anti-human-LAP-PE (BioLegend).

### Assessment of Treg functionality

PBMCs were seeded at 1 × 10^6^ cells/well in 48-well plates, stimulated with human T cell Activation/Expansion kit, in RPMI containing 10% human AB serum, penicillin/streptomycin, and 2 mM l-glutamine at 37 °C, 5% CO_2_. After 7 days, cells were stimulated again for 4 h with 50 ng/ml PMA and 1 mM ionomycin (Sigma-Aldrich) in the presence of a protein transport inhibitor (Golgi plug, BD Biosciences) containing Brefeldin A (1X). Culture supernatants were collected and kept frozen at − 80 °C until IgA quantification by ELISA (IgA human ELISA kit, Thermo Fisher Scientific).

Tregs (CD3 + /CD25hi/CD127 −) were removed from PBMCs with a MOFLO ASTRIOS cell sorter (Beckman Coulter).

### Breg lymphocytes

PBMCs were seeded at 1 × 10^6^ cells/well in 48-well plates. Cells were stimulated with recombinant human CD40 Ligand/TNFSF5 (histidine-tagged) (R&D Systems) 1 µg/ml and ODN 2006 (Invivogen) 10 µg/ml in RPMI containing 10% human AB serum, penicillin/streptomycin, and 2 mM l-glutamine at 37 °C, 5% CO_2_. After 24 h, cells were stimulated again for 4 h with 50 ng/ml PMA and 1 mM ionomycin in the presence of a Golgi plug containing Brefeldin A (1X). After stimulation, cells were stained with Zombie Green dye, anti-CD19 PC7, and anti-IL-10 PE (BioLegend) using the Intraprep Permeabilization Kit (Beckman Coulter).

### Flow cytometry

Samples were acquired on a Navios cytometer and analyzed using the Kaluza software (Beckman Coulter).

### Immunoglobulin assay

Immunoglobulin A, G, and M levels in the serum were measured by immunonephelometry (COBAS® 6000).

### Cytokine production analysis

Cytokines in the serum were measured by Luminex immunoassay (ProcartaPlex, Thermo Fisher Scientific).

### Statistical analysis

The normality of the distribution of quantitative variables was explored using the Shapiro-Wilks normality test and kurtosis and skewness coefficients. Statistical results were presented as medians and interquartile ranges.

The percentage of Tregs in each group was compared by variance analysis completed by the Holm-Bonferroni correction method to correct the significance level in multiple comparisons.

All tests were two-sided, and analyses were performed using the SAS Institute, Cary, NC, USA, version 9.4 software.

Correlation between the different variables studied was assessed by calculating the Spearman coefficient.

### Ethical approval

This study was approved by the CPP Sud Méditerranée III ethical committee, reference n°2013.10.05. Guardians of parental authority and children depending on their age gave written informed consent.

## Results

### Population characteristics

Thirty patients in group A and 30 patients in group B were compared with 40 HCs. The 3 groups were comparable according to age and sex (Table 1). The patients’ clinical characteristics are summarized in Table 2. The time between the start of the disease (first symptoms, whatever the involvement) and the inclusion visit was really short in group A (median, 4 days; Q1, 2 days; Q3, 13 days). In group B, the median time between the start of the disease and the inclusion visit was 246 days (Q1, 143 days; Q3, 417 days). In this group, the median period of time between the last symptoms of the acute phase and the inclusion visit was 154 days (Q1, 93 days; Q3, 280 days). All patients had cutaneous involvement, a majority had articular involvement, and one-third had gastrointestinal tract involvement. Forty-four percent of patients had proteinuria ≥ 30 mg/dl and/or hematuria ≥ 80 red cells/microliter on the dipstick. Platelet, WBC, neutrophil, and monocyte counts were higher in group A. Details of lymphocyte subpopulations are shown in Table 3.

**Table 1:** Demographic and clinical characteristics of the populations under study

Group	A	B	C
Number of subjects	30	30	40
Sex (F/M)	15/15	15/15	23/17
Age (years)	6.1 (4.5; 7.2)	6.6 (5.3; 9.7)	6.2 (4.65; 9.5)
Weight (kg)	18.5 (17; 25)	22.5 (18; 32)	21 (17.5; 34)
Height (cm)	112 (103; 119)	119.5 (113; 134)	119 (107.5; 138.5)
Body mass index (kg/m^2^)	16.02 (14.12; 17.73)	15.67 (14.62; 17.09)	15.56 (14.74; 17.35)
Systolic blood pressure (mmHg)	101 (95; 109)	97 (92; 107)	99.5 (93; 110.5)
Diastolic blood pressure (mmHg)	57 (53; 65)	57 (53; 63)	65 (56.5; 70)
Heart rate (bpm)	101.5 (88; 110)	90.5 (75; 101.5)	83.5 (74; 95)

**Table 2:** Main clinical characteristics of HSP in groups A and B at inclusion

		A, *n* = 30 (%)	B, *n* = 30 (%)
**Cutaneous involvement**		30 (100)	30(100)
	Palpable purpura	30	30
	Necrotic lesions	6	7
	Edema of the extremities	21	22
**Abdominal involvement**		19 (63)	22 (73)
	Abdominal pain	19	21
	Vomiting	8	12
	Diarrhea	3	2
	Anorexia	8	14
	Gastrointestinal bleeding	2	4
	Others	1	8
**Articular involvement**		27 (90)	30 (100)
	Arthralgias	20	0
	Arthritis	4	4
	Periarticular edema	21	18
	Muscle pain	9	8
**Renal involvement**			
	Proteinuria ≥ 1 + and/or hematuria ≥ 2 +	13 (45)^a^	13 (43)
	Lower back pain	1	1
	Edema	4	9
	High blood pressure	1	5
	Macroscopic hematuria	2	6
**Genital involvement**	Orchitis	5 (33)^b^	3 (20)^b^

^a^The data was missing for one patient; the total number of patients with HSP in this analysis is 29 (not 30)

^b^Percent expressed according to the male population in the group

**Table 3:** Blood count and lymphocyte subpopulations at inclusion in groups A, B, and C

Patient groups	A	B	C	*p* value global	Holm adjusted *p* value		
					A vs. B	A vs. C	B vs. C
Number of subjects	30	30	40				
Hemoglobin, g/dl (1/0/13)^a^	12.3 [11.8; 12.9]	12.75 [12.1; 13.3]	12.6 [11.9; 13.2]	0.2421	0.2159	0.5795	0.5795
Platelets/mm^3^ (1/0/13)^a^	367 [323; 410]	313.5 [274; 335]	295 [255; 367]	**0.0002**	**0.0003**	**0.0003**	0.8925
WBC/mm^3^ (1/0/13)^a^	9.2 [6.61; 12.16]	7.55 [6.3; 8.78]	6.81 [5.93; 8.3]	**0.0153**	**0.0103**	**0.0103**	0.9485
Neutrophils/mm^3^ (3/0/13)^a^	4.66 [3.49; 6.59]	3.44 [2.47; 4.42]	3.31 [2.25; 4.64]	**0.0133**	**0.0069**	**0.0103**	0.8435
Monocyte/mm^3^ (3/0/13)^a^	0.68 [0.52; 0.99]	0.59 [0.47; 0.7]	0.52 [0.44; 0.6]	**0.0174**	0.9187	0.6018	0.6018
Lymphocytes/mm^3^ (3/0/13)^a^	2.85 [1.99; 3.94]	2.72 [2.26; 3.4]	2.76 [2.29; 3.42]	0.6895	1.0	1.0	1.0
CD3^+^/mm^3^ (2/3/3)^a^	1440 [1179; 1958.5]	1828 [1504; 2534]	1458 [1184; 2074]	0.1329	0.3510	0.9476	0.3510
% CD3^+^ (1/1/3)^a^	74.29 [68.16; 77.7]	75.44 [70.54; 79.54]	74.02 [70.25; 77.15]	0.5626	0.7962	0.7962	0.5803
CD4^+^/mm^3^ (2/3/4)^a^	901.5 [666; 1187.5]	1136 [795; 1335]	897 [672.5; 1312]	0.2268	0.4205	0.8133	0.4205
% CD4^+^ (1/1/4)^a^	41.49 [38.35; 47.69]	44.78 [38.57; 49.52]	45.57 [40.85; 50.15]	0.4827	1.0	1.0	1.0
CD8^+^/mm^3^ (2/3/4)^a^	458 [395; 666.5]	568 [477; 783]	515 [379.5; 668]	0.2212	0.7008	0.7008	0.5113
% CD8^+^ (1/1/4)^a^	22.45 [19.86; 29.96]	24.91 [21.69; 29.9]	24.29 [21.78; 25.87]	0.5112	1.0	1.0	1.0
CD19^+^/mm^3^ (2/4/4)^a^	351 [253.5; 583]	419 [210; 512]	364.5 [273; 449]	0.9401	1.0	1.0	1.0
% CD19^+^ (1/2/4)^a^	18 [14.48; 21.02]	15.63 [12.52; 19.19]	17.37 [13.01; 21.21]	0.3043	0.2898	0.9408	0.2898
CD3^−^CD56^+^/mm^3^ (2/4/4)^a^	78 [54; 143]	122.5 [71; 219]	88 [69.5; 129]	0.3068	0.6492	0.8993	0.6492
% CD3^−^CD56^+^ (1/2/4)^a^	4.23 [2.71; 5.77]	4.86 [2.7; 8.26]	4.31 [2.66; 6.37]	0.8390	1.0	1.0	1.0
Tregs/mm^3^ (4/4/8)^a^	50.01 [35.09; 84.48]	50.53 [29.1; 68.44]	39.44 [20.95; 73.82]	0.3871	0.4678	0.3192	0.7101
% Tregs (3/1/4)^a^	6.53 [4.24; 9.21]	4.33 [3.6; 5.66]	4.45 [3.01; 6.6]	**0.0049**	**0.0016**	**0.0029**	0.6253
% CD4^+^CD25^+^ (3/5/7)^a^	6.72 [5.47; 9.16]	5.96 [4.51; 7.64]	5.72 [4.6; 9.32]	0.3876	1.0000	1.0000	1.0000
% CD4^+^ central memory (3/7/7)^a^	15.13 [8.98; 22.59]	13.38 [7.58; 20.67]	9.72 [7.19; 13.76]	0.0899	0.4043	**0.0356**	0.4043
% CD4^+^ effector memory (3/7/7)^a^	16.38 [11.77; 21.97]	14.17 [8.78; 17.42]	15.79 [11.68; 19.4]	0.3624	1.0000	1.0000	1.0000
% CD4^+^ naives (3/7/7)^a^	58.47 [48.59; 64.28]	61.65 [56.15; 64.71]	61.78 [54.65; 64.29]	0.3546	0.7739	0.5977	0.7739
% CD8^+^ central memory (3/7/6)^a^	5.78 [4.08; 11.75]	7.14 [3.96; 12.15]	7.61 [5.07; 10.12]	0.9066	1.0000	1.0000	1.0000
% CD8^+^ effector memory (3/7/6)^a^	8.39 [5.15; 11.98]	8.44 [3.72; 15.83]	10.5 [6.06; 15.83]	0.3803	0.7549	0.4377	0.5732
% CD8^+^ naives (3/7/6)^a^	67.55 [58.77; 75.82]	68.43 [57.08; 75.67]	67.68 [54.28; 74.17]	0.5280	0.8284	0.4365	0.4871
% Bregs (11/6/17)^a^	6.47 [5.34; 8.70]	5.86 [4.45; 9.27]	6.90 [5.53; 9.39]	0.7001	1.0000	1.0000	1.0000

Data are shown as median (interquartile range q1; q3)

*CM* Central memory, *EM* Effector memory, *tEM* Terminally differentiated, *SM* Switched memory, *NSM* Non-switched memory, *DN* Double-negative

^a^Missing data respectively for n patients in groups A, B, and C

### Treg, Th3, and Breg cell frequencies in HSP

**Fig. 1:**
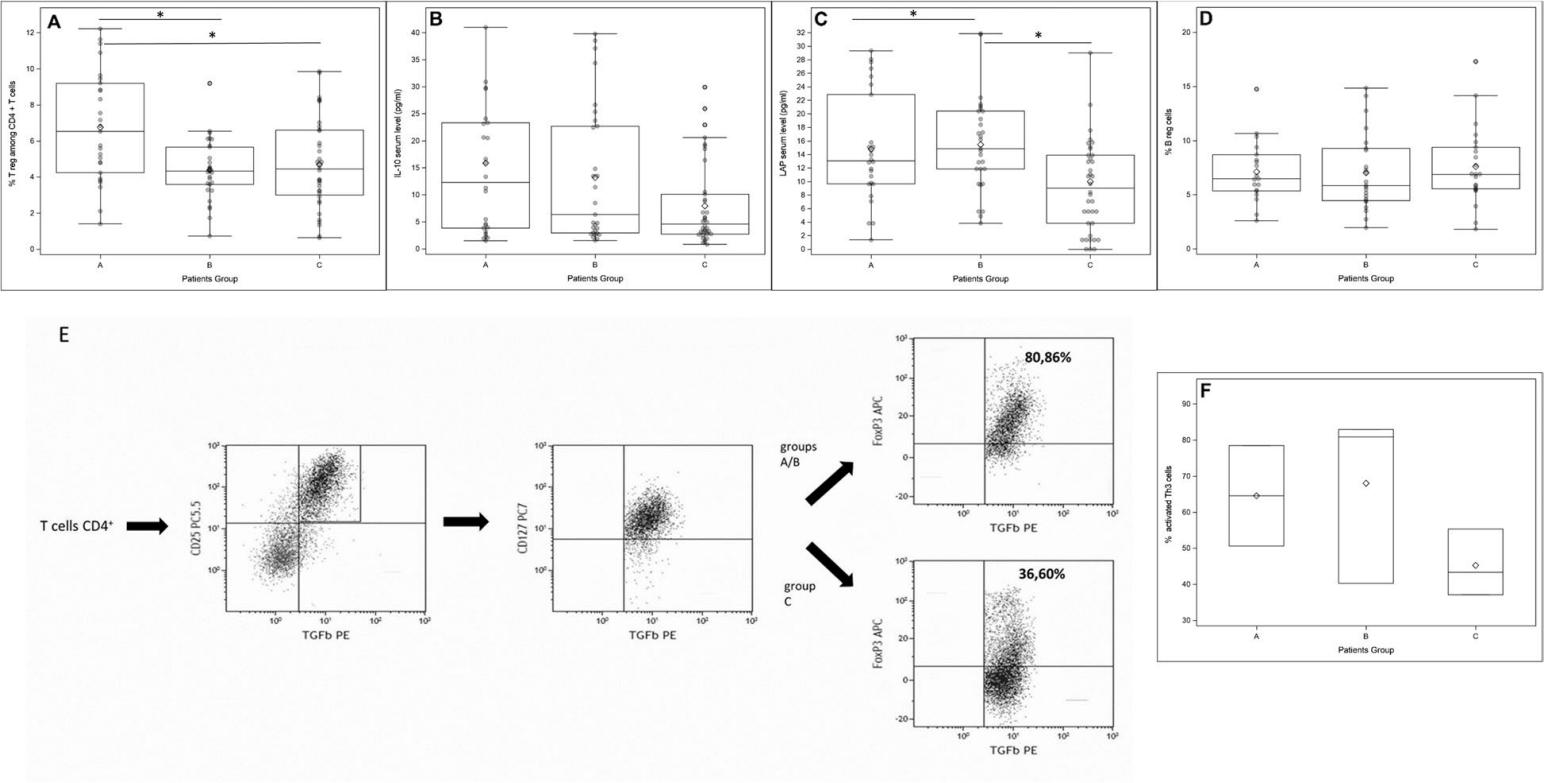
Frequencies of Treg (**A**), Breg (**D**), IL-10 (**B**), and LAP (**C**) serum levels, group A, B, or C. Study of Th3 activation, groups A, B, or C (2, 3, and 3 for groups A, B, and C, respectively) (**E**, **F**). The percentage of activated Th3 cells corresponds to percentages of CD4 + T cells producing LAP and FoxP3 + . *p value < 0.05

Patients in the acute phase had a higher percentage of Tregs than patients on remission (6.53% [4.24; 9.21] vs. 4.33% [3.6; 5.66], *p* = 0.002) and HCs (6.53% [4.24; 9.21] vs. 4.45% [3.01; 6.6], *p* = 0.003) (Table 3, Fig. 1A). In line with the high proportion of Tregs observed in acute HSP, IL-10 serum levels tended to be higher in acute than in remittent HSP (12.32 pg/ml [3.86; 23.33] vs. 6.36 pg/ml [2.95; 22.68], *p* = 0.089) or HCs (4.59 pg/ml [2.76; 10.1], *p* = 0.403) (Fig. 1B, Additional file 1: Table S1).

We then wondered whether another regulatory T cell subpopulation, Th3 cells, capable of producing the anti-inflammatory cytokine TGF-beta, was more abundant in the acute phase group than in the other groups. We had enough PBMCs from certain children of each group (*n* = 2, 3, 3 for groups A, B, and C, respectively) to stimulate them with a mitogen and measure the percentages of CD4 + T cells producing latency-associated peptide (LAP), a marker of TGF-beta production (Fig. 1E). Th3 cells were not more abundant in HSP patients, but activated Th3 cells (i.e., Th3 FoxP3 + cells) tended to be more abundant in the acute and remittent phases than in HCs (78.43% [50.62; 80.84] vs. 43.30% [40.20; 49.32], *p* = 0.135) (Fig. 1F). Accordingly, LAP serum levels were higher in group A (13.08 pg/ml [9.64; 22.87], *p* = 0.003) and group B (14.86 pg/ml [11.88; 20.41], *p* = 0.040) than in group C (9.03 pg/ml [3.82; 13.91]) (Fig. 1C, Additional file 1: Table S1).

Breg cells (B cells able to produce IL-10 under stimulation) were also quantified. We found no difference (6.47% [5.38; 8.70], 5.86% [4.45; 9.27], and 6.90% [5.53; 9.39], for groups A, B, and C, respectively), *p* = 0.700 (Fig. 1D, Additional file 1: Table S1).

### Inflammatory cytokines are higher in HSP at the acute phase

**Fig. 2:**
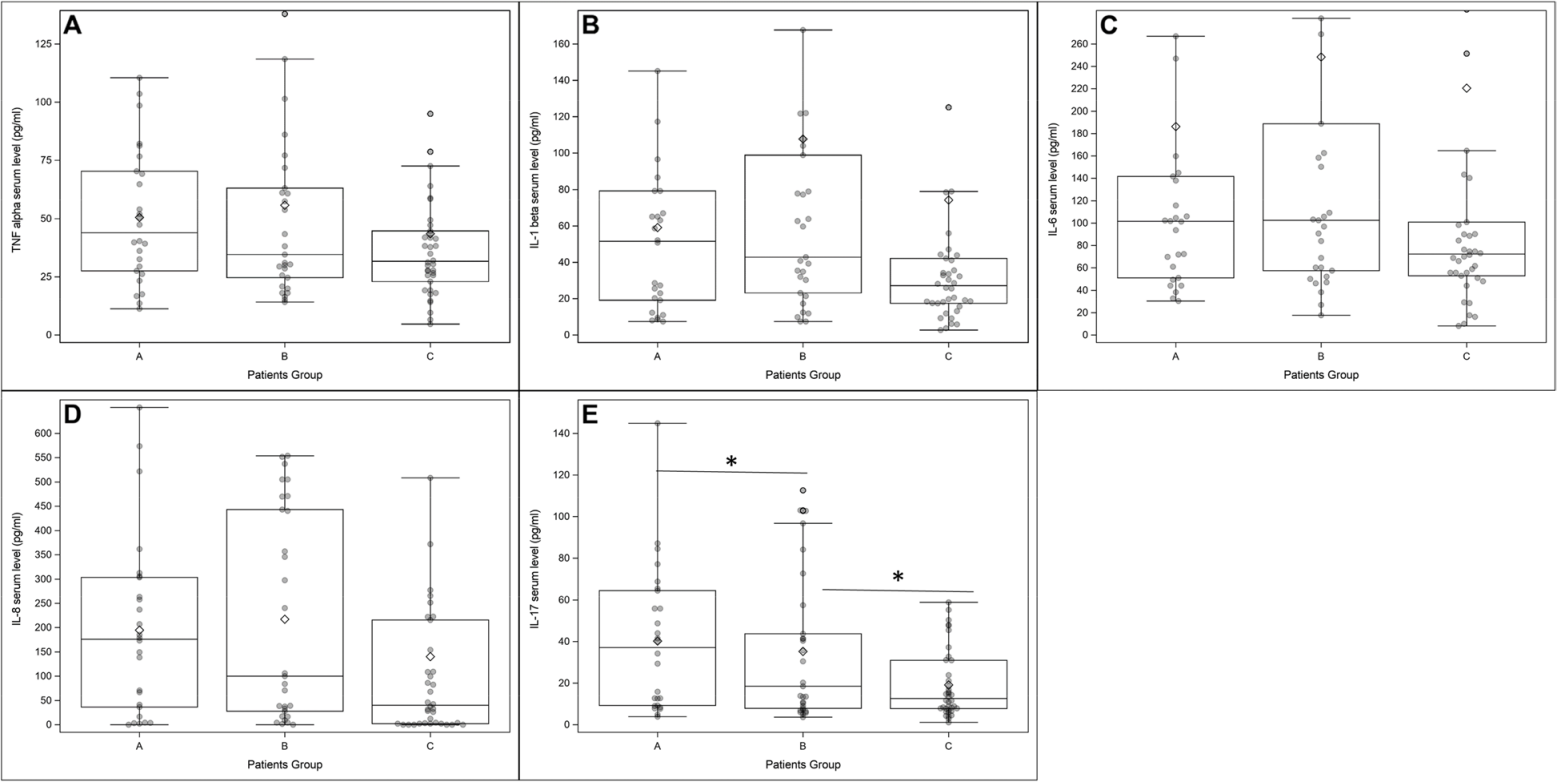
TNF-alpha (**A**), IL-1-beta (**B**), IL-6 (**C**), IL-8 (**D**), and IL-17 (**E**) serum levels, groups A, B, and C. *p value < 0.05

To test whether a higher percentage of Tregs and Th3 in group A resulted in efficient control of inflammation, we measured the serum concentrations of inflammatory cytokines in all groups (Fig. 2, Additional file 1: Table S1). TNF-alpha, IL-1beta, and IL-6 levels tended to be higher in group A than in the other groups.

IL-17A levels (37.16 pg/ml [9.21; 64.5] vs. 18.47 pg/ml [7.84; 43.7] vs. 12.66 pg/ml [7.74; 31]) were significantly higher in group A than in group B (*p* = 0.042) and in group B compared to group C (*p* = 0.014).

### Immune regulation in patients with HSP nephritis

**Fig. 3:**
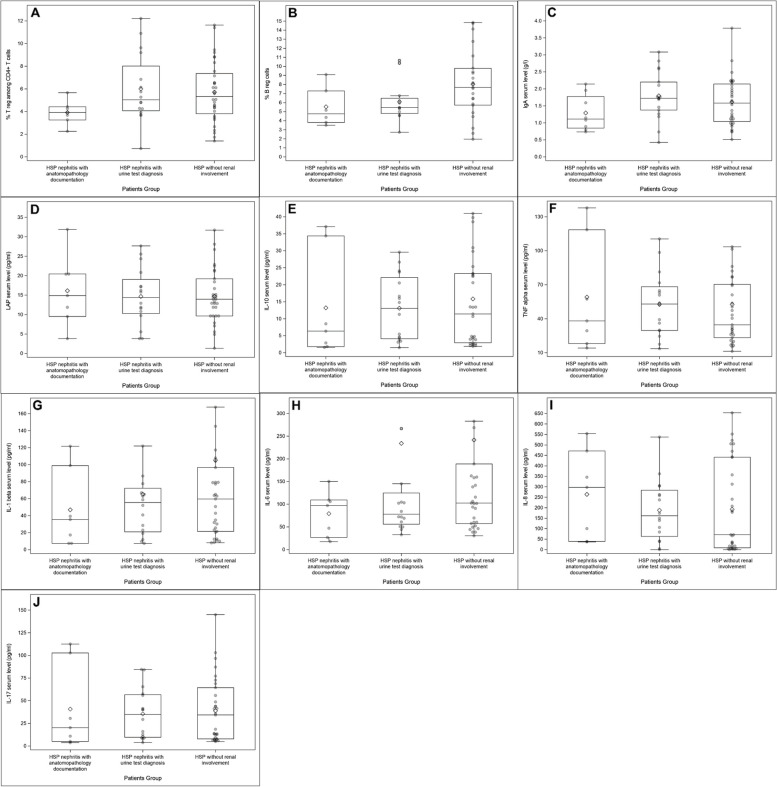
Frequencies of Treg (**A**), Breg (**B**), IgA (**C**), LAP (**D**), IL-10 (**E**), TNF-alpha (**F**), IL-1-beta (**G**), IL-6 (**H**), IL-8 (**I**), and IL-17 (**J**) serum levels according to group: HSP patients with nephritis with anatomopathological documentation (HSPNb) or nephritis with urine test diagnosis (HSPNu) or without renal involvement (HSPw). *p value < 0.05

Results of the analyses of regulatory immune cells and soluble factors in HSP patients with nephritis (HSPNb and HSPNu) or without renal involvement (HSPw) are presented in Fig. 3 and Additional file 2: Table S2.

Comparing HSPNb, HSPNu, and HSPw groups respectively, Treg frequencies were not different between the groups (3.92% [3.26; 4.43] vs. 5.05% [4.08; 8.02] vs. 5.33% [3.81; 7.36], *p* = 0.133). However, the percentage of Bregs tended to be lower in HSP nephritis (HSPNb or HSPNu) compared to HSP without renal involvement (4.76% [3.82; 7.30] vs. 5.45% [4.80; 6.48] vs. 7.67% [5.73; 9.80], *p* = 0.101).

In the serum, LAP and IL-10 levels were not different between the groups (Additional file 2: Table S2). Results for IL-1beta, IL-17A, IL-6, IL-8, and TNF-alpha are detailed in Fig. 3 and Additional file 2: Table S2. No results were statistically significant.

### IgA dysregulation in HSP

**Fig. 4:**
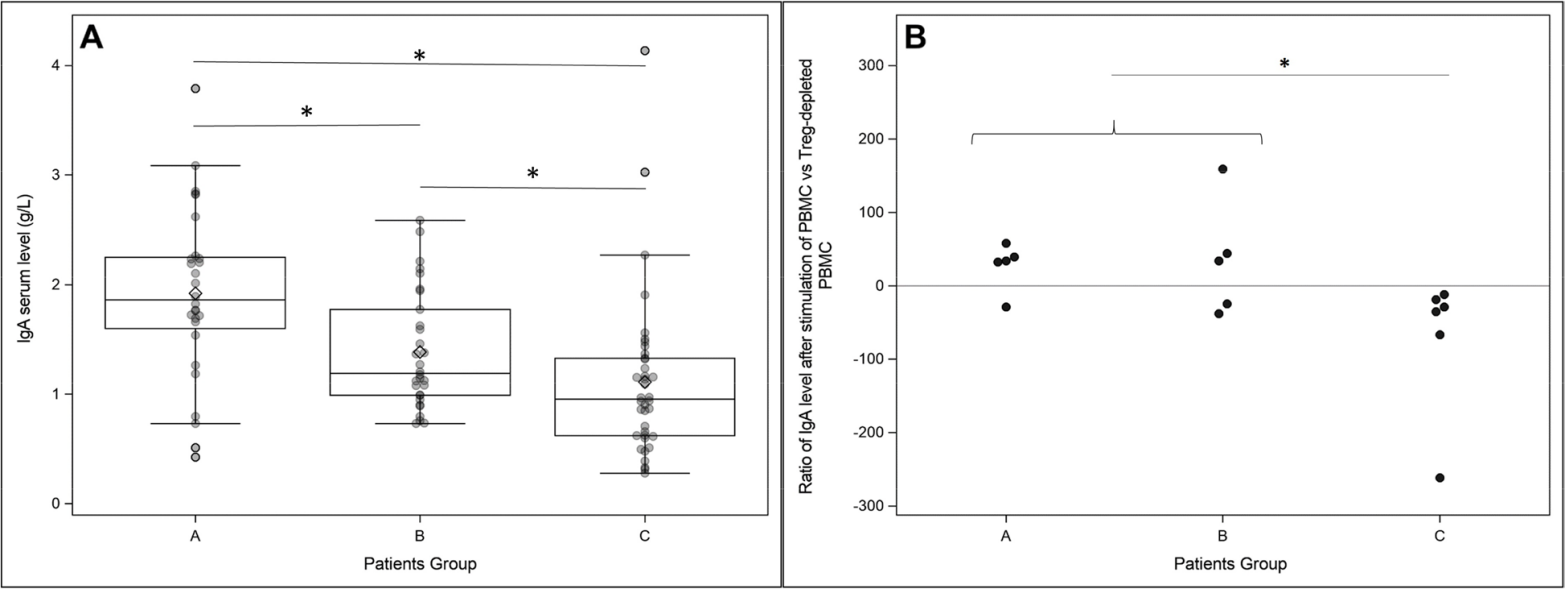
IgA serum levels, groups A, B, and C (**A**) and effect of prior Treg depletion on the emergence of IgA-secreting cells (**B**). Ratio of IgA level after stimulation of PBMCs vs. Treg-depleted PBMCs. *p value < 0.05

Serum IgA levels were significantly higher in group A (1.86 g/L [1.6; 2.25]) than in group B (1.19 g/L [0.99; 1.77], *p* < 0.0001) and HCs (0.95 g/L [0.62; 1.33], *p* = 0.010) (Fig. 4A, Additional file 1: Table S1). Serum IgG and IgM levels were similar in all three groups.

IL-2, IL-4, IL-5, IL-6, IL-10, IL-17, and especially TGF-beta favor IgA synthesis [26–28]. Therefore, Treg and Th3 expansion may result in IgA overproduction. To assess the role of Tregs in IgA switch, we evaluated the effect of prior Treg depletion on the emergence of IgA-secreting cells among patients PBMCs under polyclonal stimulation (*n* = 5, 6, and 6 for A, B, and C groups, respectively). In HCs, Treg depletion reduced the ability of activated PBMCs (− 32.20% [− 58.83; − 21.50]) to differentiate into IgA-producing cells (Fig. 4B). By contrast, Treg removal resulted in an increase in IgA concentration in the patients PBMC supernatant (33.7% [32.22; 39.00] and 39.0% [− 9.93; 130.7] respectively for groups A and B, *p* [(A + B) vs. C] = 0.015) (Fig. 4B). Thus, in HSP patients, the effect of Treg on IgA switch is the opposite of the effect observed in HCs: Treg cells downregulated IgA overproduction in HSP patients. Accordingly, Treg percentage and blood IgA concentration tended to be negatively correlated in groups A and B (*r* =  − 0.2) but positively correlated in HCs (*r* = 0.4).

## Discussion

The involvement of Tregs in HSP has often been suspected. By contrast to the literature data, our work highlighted that a higher percentage of Tregs was associated with HSP. Indeed, previous studies described an absence of difference in Treg percentages between HSP and HCs [29], or even a decrease in Treg percentages in HSP compared to HCs [25, 30, 31].

In adults, age, sex, and ethnicity have emerged as major factors contributing to variations in lymphocyte phenotype composition [32, 33]. For example, the reference range of Tregs proposed for adult Chinese and Italian populations is different (2.17–7.94% vs. 0.59–0.79%). In childhood, Treg percentages are similar in the male and female groups (personal data not shown). The absolute number of lymphocytes drastically decreases with age with a significant slope in both male and female groups. However, there is no correlation between Treg percentages and age (personal data not shown).

One explanation for these divergent data may be how Tregs are identified [34, 35]. Another possibility is the clinical forms of the disease analyzed. The timing of sampling relative to the onset of disease may also influence the results of immune parameters. In two of three children, there are no recurrent episodes [1]. Furthermore, one retrospective study found no biological differences between patients with only one HSP flare and those with HSP recurrence [36].

In our HSP population, there was an increase in the IL-17A serum level and a similar trend for IL-1beta and IL-8 serum levels, as reported earlier [29, 31, 37]. This suggests that the action of Treg and Th3 is insufficient to control inflammation.

In our population, Breg percentages tended to be lower in HSP nephritis compared to HSP without kidney involvement. Previously, in HSP children compared to HCs, Yang et al. found a decrease in Breg frequencies in HSP with kidney impairment compared to HSP without kidney impairment and HCs, and no difference in Breg frequencies between HSP on remission and HCs [25]. In an adult population with non-treated HSP nephritis compared to HCs, Breg frequencies and IL-10 levels appeared lower, yet, on treatment, both parameters were restored [24]. This leads to the interesting hypothesis that Bregs might play a role in preventing nephritis in HSP.

As already described, we found an increase in serum IgA levels [9]. In our study, like others [9, 20, 37], serum TGF-beta levels were higher in the HSP population. Li et al. described a tendency towards an increase in TGF-beta levels [29]. This is in line with the fact that TGF-beta induces an IgA switch [17–19]. Indeed, mice deficient in TGF-beta or receptor II TGF-beta have low levels of IgA [38, 39].

A striking observation in our study is the paradoxical effect of patients’ Tregs on IgA-secreting cells in vitro. Physiologically, Tregs promote an IgA switch. Thus, in mice, Treg depletion reduces circulating IgA levels, and the transfer of Tregs promotes IgA production via TGF-beta [26, 27]. The effect of Tregs on IgA is the same in IgA nephropathy, since IgA serum levels of rats that received Tregs from patients with an IgA nephropathy were significantly higher than in rats that received Tregs from a control group [40]. In the HCs of our study, Treg depletion did reduce IgA production. However, we observed that HSP patients’ Tregs depletion favored IgA production. Our data are in line with the inverse correlation between the number of circulating Tregs and serum IgA concentrations noted in patients with ankylosing spondylitis [41]. This suggests that, in HSP, Tregs might be trying to dampen IgA synthesis rather than induce it. One hypothesis is that activated Th3 cells are responsible for an IgA overproduction/switch that Tregs try to dampen. Another hypothesis is that it is the response of immunoglobulin A-secreting cells to Tregs which is modified in HSP. Further studies are needed to identify whether this is a regulatory deficit due to Tregs or to the response to Tregs.

Based on these results, we propose the following regulatory pattern for HSP (Additional file 3: Fig. S1): Following immune stimulation by a potential viral or bacterial infection [15], the antigen-presenting cells activate the Th3 cells which, by secreting TGF-beta, lead to IgA overproduction by B cell lineage. The IgA produced are deposited on the vessels leading to vasculitis and tissue damage. The Tregs which are not deficient try to dampen the inflammation and, surprisingly, IgA production during the acute phase. In patients with Breg deficiency, the uncontrolled production and deposition of probably abnormally glycosylated IgA [10] will lead to kidney damage.

## Conclusion

To summarize, we observed an increase in Tregs and Th3 cells apparently failing to inhibit immune activation. Remarkably, Breg cells are fewer in the HSP population with nephropathy. Finally, we unveiled the unusual negative effect of Tregs on IgA production which is also insufficient.

### Supplementary Information

**Additional file 1: Table S1.** Biological data, Groups A, B and C.

**Additional file 2: Table S2.** Biological data according to whether patients have HSP nephritis or not.

**Additional file 3: Fig. S1.** Model showing the role of regulatory B and T cells in pediatric HSP.

## Data Availability

The datasets generated during and/or analyzed during the current study are available from the corresponding author upon reasonable request.

## Abbreviations

Bregs: Regulatory B cells
EULAR: European League Against Rheumatism
HCs: Healthy controls
HLA: Human leukocyte antigen
HSP: Henoch–Schönlein purpura
HSPNb: HSP patients with biopsy documented nephritis
HSPNu: HSP nephritis patients with urine test diagnosis
HSPNw: HSP patients without renal involvement
IgA: Immunoglobulin A
LAP: Latency-associated peptide
NK: Natural killer cells
PBMCs: Peripheral blood mononuclear cells
PMA: Phorbol 12-myristate 13-acetate
PRES: Pediatric Rheumatology European Society
PRINTO: Pediatric Rheumatology International Trials Organization
SLE: Systemic lupus erythematosus
TGF-beta: Transforming growth factor-β
Th3: Helper T3 cells
Tregs: Regulatory T cells

## References

[1] Saulsbury FT. Henoch-Schonlein purpura in children. Report of 100 patients and review of the literature. Medicine (Baltimore) 1999. 10.1097/00005792-199911000-00005 10.1097/00005792-199911000-00005 10575422

[2] Trapani S, Micheli A, Grisolia F, Resti M, Chiappini E, Falcini F et al. Henoch Schonlein purpura in childhood: epidemiological and clinical analysis of 150 cases over a 5-year period and review of literature. Semin Arthritis Rheum.2005. 10.1016/j.semarthrit.2005.08.007. 10.1016/j.semarthrit.2005.08.007 16325655

[3] Yang YH, Hung CF, Hsu CR, Wang LC, Chuang YH, Lin YT et al. A nationwide survey on epidemiological characteristics of childhood Henoch-Schonlein purpura in Taiwan.Rheumatology (Oxford). 2005. 10.1093/rheumatology/keh544. 10.1093/rheumatology/keh544 15671050

[4] Gardner-Medwin JM, Dolezalova P, Cummins C, Southwood TR. Incidence of Henoch-Schonlein purpura, Kawasaki disease, and rare vasculitides in children of different ethnic origins. Lancet. 2002. 10.1016/S0140-6736(02)11279-7. 10.1016/S0140-6736(02)11279-7 12401245

[5] Aalberse J, Dolman K, Ramnath G, Pereira RR, Davin JC (2007). Henoch Schonlein purpura in children: an epidemiological study among Dutch paediatricians on incidence and diagnostic criteria. Ann Rheum Dis.

[6] Yang YH, Yu HH, Chiang BL (2014). The diagnosis and classification of Henoch-Schonlein purpura: an updated review. Autoimmun Rev.

[7] Ozen S, Pistorio A, Iusan SM, Bakkaloglu A, Herlin T, Brik R et al. EULAR/PRINTO/PRES criteria for Henoch-Schonlein purpura, childhood polyarteritis nodosa, childhood Wegener granulomatosis and childhood Takayasu arteritis: Ankara 2008. Part II: Final classification criteria. Ann Rheum Dis 2010; doi:10.1136/ard.2009.116657 10.1136/ard.2009.116657 20413568

[8] Kawasaki Y (2011). The pathogenesis and treatment of pediatric Henoch-Schonlein purpura nephritis. Clin Exp Nephrol.

[9] Saulsbury FT (2007). Clinical update: Henoch-Schonlein purpura. Lancet.

[10] Davin JC, Coppo R (2014). Henoch-Schonlein purpura nephritis in children. Nat Rev Nephrol.

[11] Brendel-Muller K, Hahn A, Schneppenheim R, Santer R (2001). Laboratory signs of activated coagulation are common in Henoch-Schonlein purpura. Pediatr Nephrol.

[12] Sestan M, Kifer N, Sozeri B, Demir F, Ulu K, Silva CA (2023). Clinical features, treatment and outcome of pediatric patients with severe cutaneous manifestations in IgA vasculitis: multicenter international study. Semin Arthritis Rheum.

[13] Vaahtovuo J, Munukka E, Korkeamaki M, Luukkainen R, Toivanen P. Fecal microbiota in early rheumatoid arthritis. J Rheumatol 2008; 18528968

[14] Yeoh N, Burton JP, Suppiah P, Reid G, Stebbings S (2013). The role of the microbiome in rheumatic diseases. Curr Rheumatol Rep.

[15] Rigante D, Castellazzi L, Bosco A, Esposito S (2013). Is there a crossroad between infections, genetics, and Henoch-Schonlein purpura?. Autoimmun Rev.

[16] Barzaghi F, Passerini L (2021). IPEX syndrome: improved knowledge of immune pathogenesis empowers diagnosis. Front Pediatr.

[17] Coffman RL, Lebman DA, Shrader B (1989). Transforming growth factor beta specifically enhances IgA production by lipopolysaccharide-stimulated murine B lymphocytes. J Exp Med.

[18] Sonoda E, Matsumoto R, Hitoshi Y, Ishii T, Sugimoto M, Araki S (1989). Transforming growth factor beta induces IgA production and acts additively with interleukin 5 for IgA production. J Exp Med.

[19] van Vlasselaer P, Punnonen J, de Vries JE. Transforming growth factor-beta directs IgA switching in human B cells. J Immunol. 1992. 1347548

[20] Yang YH, Huang MT, Lin SC, Lin YT, Tsai MJ, Chiang BL (2000). Increased transforming growth factor-beta (TGF-beta)-secreting T cells and IgA anti-cardiolipin antibody levels during acute stage of childhood Henoch-Schonlein purpura. Clin Exp Immunol.

[21] Anolik JH, Barnard J, Owen T, Zheng B, Kemshetti S, Looney RJ (2007). Delayed memory B cell recovery in peripheral blood and lymphoid tissue in systemic lupus erythematosus after B cell depletion therapy. Arthritis Rheum.

[22] Mauri C, Gray D, Mushtaq N, Londei M (2003). Prevention of arthritis by interleukin 10-producing B cells. J Exp Med.

[23] Correale J, Farez M, Razzitte G (2008). Helminth infections associated with multiple sclerosis induce regulatory B cells. Ann Neurol.

[24] Hu X, Tai J, Qu Z, Zhao S, Zhang L, Li M (2016). A lower proportion of regulatory B cells in patients with Henoch-Schoenlein purpura nephritis. PLoS ONE.

[25] Yang B, Tan X, Xiong X, Wu D, Zhang G, Wang M (2017). Effect of CD40/CD40L signaling on IL-10-producing regulatory B cells in Chinese children with Henoch-Schonlein purpura nephritis. Immunol Res.

[26] Cong Y, Feng T, Fujihashi K, Schoeb TR, Elson CO (2009). A dominant, coordinated T regulatory cell-IgA response to the intestinal microbiota. Proc Natl Acad Sci U S A.

[27] Feng T, Elson CO, Cong Y (2011). Treg cell-IgA axis in maintenance of host immune homeostasis with microbiota. Int Immunopharmacol.

[28] Cerutti A, Rescigno M (2008). The biology of intestinal immunoglobulin A responses. Immunity.

[29] Li YY, Li CR, Wang GB, Yang J, Zu Y (2012). Investigation of the change in CD4(+) T cell subset in children with Henoch-Schonlein purpura. Rheumatol Int.

[30] Chen O, Zhu XB, Ren H, Wang YB, Sun R (2013). The imbalance of Th17/Treg in Chinese children with Henoch-Schonlein purpura. Int Immunopharmacol.

[31] Li B, Ren Q, Ling J, Tao Z, Yang X, Li Y (2019). The change of Th17/Treg cells and IL-10/IL-17 in Chinese children with Henoch-Schonlein purpura: a PRISMA-compliant meta-analysis. Medicine (Baltimore).

[32] Niu HQ, Zhao XC, Li W, Xie JF, Liu XQ, Luo J (2020). Characteristics and reference ranges of CD4(+)T cell subpopulations among healthy adult Han Chinese in Shanxi province. North China BMC Immunol.

[33] Sorrenti V, Marenda B, Fortinguerra S, Cecchetto C, Quartesan R, Zorzi G (2016). Reference values for a panel of cytokinergic and regulatory lymphocyte subpopulations. Immune Netw.

[34] Sakaguchi S, Miyara M, Costantino CM, Hafler DA (2010). FOXP3+ regulatory T cells in the human immune system. Nat Rev Immunol.

[35] Miyara M, Yoshioka Y, Kitoh A, Shima T, Wing K, Niwa A (2009). Functional delineation and differentiation dynamics of human CD4+ T cells expressing the FoxP3 transcription factor. Immunity.

[36] Prais D, Amir J, Nussinovitch M (2007). Recurrent Henoch-Schonlein purpura in children. J Clin Rheumatol.

[37] Jen HY, Chuang YH, Lin SC, Chiang BL, Yang YH (2011). Increased serum interleukin-17 and peripheral Th17 cells in children with acute Henoch-Schonlein purpura. Pediatr Allergy Immunol.

[38] Cazac BB, Roes J (2000). TGF-beta receptor controls B cell responsiveness and induction of IgA in vivo. Immunity.

[39] Borsutzky S, Cazac BB, Roes J, Guzman CA (2004). TGF-beta receptor signaling is critical for mucosal IgA responses. J Immunol.

[40] Huang H, Peng Y, Long XD, Liu Z, Wen X, Jia M (2013). Tonsillar CD4+CD25+ regulatory T cells from IgA nephropathy patients have decreased immunosuppressive activity in experimental IgA nephropathy rats. Am J Nephrol.

[41] Zhao SS, Hu JW, Wang J, Lou XJ, Zhou LL (2011). Inverse correlation between CD4+ CD25high CD127low/- regulatory T-cells and serum immunoglobulin A in patients with new-onset ankylosing spondylitis. J Int Med Res.

