# Supplementary material for: Regulatory T and B cells in pediatric Henoch–Schönlein purpura: friends or foes?
Source: Arthritis Res Ther. 2024 Feb 16;26:52. doi: 10.1186/s13075-024-03278-w (PMC10870453; doi:10.1186/s13075-024-03278-w)
Supplement: Supplementary file 1 — Additional file 1: Table S1. Biological data, Groups A, B and C. [file 13075_2024_3278_MOESM1_ESM.docx]

**Supplementary data. Table 1. Biological data, Groups A, B and C**

| **Group** | **A** | **B** | **C** | **Global p value** | **Holm adjusted p value** | | |
| --- | --- | --- | --- | --- | --- | --- | --- |
|  |  |  |  |  | A  *vs*  B | A  *vs*  C | B  *vs*  C |
| Number of subjects | 30 | 30 | 40 |  |  |  |  |
| % Tregs (3/1/4)^#^ | 6.53 [4.24;9.21] | 4.33 [3.6;5.66] | 4.45 [3.01;6.6] | **0.0049** | **0.0049** | **0.0016** | **0.0029** |
| Tregs/mm^3^ (4/4/8)^#^ | 50.01 [35.09;84.48] | 50.53 [29.1;68.44] | 39.44 [20.95;73.82] | 0.3871 | 0.3871 | 0.4678 | 0.3192 |
| % Bregs (11/6/17)^#^ | 6.47 [5.34 ; 8.70] | 5.86 [4.45 ; 9.27] | 6.90 [5.53 ; 9.39] | 0.7001 | 1.0000 | 1.0000 | 1.0000 |
| IgA (g/L) (2/0/2)^#^ | 1.86 [1.6;2.25] | 1.19 [0.99;1.77] | 0.95 [0.62;1.33] | **<0.001** | **<.0001** | **0.0096** | **<.0001** |
| IgG (g/L) (2/0/3)^#^ | 10.17 [7.62;12.38] | 8.88 [7.34;10.46] | 7.85 [6.44;10.59] | 0.07 | 0.0771 | 0.3206 | 0.0631 |
| IgM (g/L) (2/0/2)^#^ | 0.89 [0.66;0.99] | 0.86 [0.65;1.11] | 0.82 [0.69;1.12] | 0.98 | 0.9760 | 1.0000 | 1.0000 |
| Sum of IgA, IgG and IgM (g/L) (2/0/3)^#^ | 12.81 [9.71;16.33] | 10.94 [9.55;13.64] | 9.61 [8.27;12.64] | **0.02** | **0.0179** | 0.1575 | **0.0133** |
| IL-1-beta (pg/ml) (4/1/2)^#^ | 51.47 [19.11;79.26] | 42.81 [23.15;98.97] | 27.12 [17.49;42.14] | **0.05** | 0.0512 | 0.9339 | 0.9339 |
| IL-10 (pg/ml) (4/1/2)^#^ | 12.32 [3.86;23.33] | 6.36 [2.95;22.68] | 4.59 [2.76;10.1] | 0.09 | 0.0889 | 0.4032 | 0.0291 |
| IL-17A (pg/ml) (4/1/2)^#^ | 37.16 [9.21;64.5] | 18.47 [7.84;43.7] | 12.66 [7.74;31] | **0.04** | **0.0421** | 0.5143 | **0.0139** |
| IL-6 (pg/ml) (4/1/2)^#^ | 101.59 [51.02;141.61] | 102.59 [57.52;188.74] | 72.39 [52.91;100.95] | 0.29 | 0.2870 | 1.0000 | 1.0000 |
| IL-8 (pg/ml) (4/1/2)^#^ | 176.11 [36.23;303.28] | 100.25 [28.1;442.9] | 39.98 [2.36;215.63] | **0.05** | 0.0534 | 0.7027 | 0.6547 |
| LAP (pg/ml) (4/1/2)^#^ | 13.08 [9.64;22.87] | 14.86 [11.88;20.41] | 9.03 [3.82;13.91] | **0.003** | **0.0033** | 0.7653 | **0.0395** |
| TNF-alpha (pg/ml) (4/1/2)^#^ | 43.89 [27.52;70.31] | 34.63 [24.62;63.08] | 31.66 [23;44.73] | 0.22 | 0.2186 | 1.0000 | 1.0000 |

Data are shown as median (interquartile range q1;q3). ^#^ Missing data respectively for n patients in Groups A, B and C.
