# Supplementary material for: Regulatory T and B cells in pediatric Henoch–Schönlein purpura: friends or foes?
Source: Arthritis Res Ther. 2024 Feb 16;26:52. doi: 10.1186/s13075-024-03278-w (PMC10870453; doi:10.1186/s13075-024-03278-w)
Supplement: Supplementary file 2 — Additional file 2: Table S2. Biological data according to whether patients have HSP nephritis or not. [file 13075_2024_3278_MOESM2_ESM.docx]

**Supplementary data. Table 2. Biological data according to whether patients have HSP nephritis or not**

| **Group** | **HSP nephritis with anatomopathological documentation** | **HSP nephritis with urine test diagnosis** | **HSP without renal involvement** | **Global p value** | **Holm adjusted p value** | | |
| --- | --- | --- | --- | --- | --- | --- | --- |
|  |  |  |  |  | HSP nephritis with anatomopathological documentation  *vs.*  HSP without renal involvement | HSP nephritis with anatomopathological documentation  *vs.*  HSP nephritis with urine test diagnosis | HSP nephritis with urine test diagnosis  *vs.*  HSP without renal involvement |
| Number of subjects | 8^a^ | 18^a^ | 33^a^ |  |  |  |  |
| % Tregs(1/2/1)^#^ | 3.92 [3.26 ; 4.43] | 5.05 [4.08 ; 8.02] | 5.33 [3.81;7.36] | 0.1333 | 0.2696 | 0.2696 | 0.7183 |
| Tregs/mm3 (1/3/4)^#^ | 43.75 [38.58 ; 68.44] | 44.75 [35.09 ; 95.87] | 60.37 [32;78.65] | 0.9350 | 1.0000 | 1.0000 | 1.0000 |
| % Bregs (2/6/9)^#^ | 4.76 [3.82 ; 7.3] | 5.45 [4.80 ; 6.48] | 7.67 [5.73;9.80] | 0.1009 | 0.7318 | 0.2754 | 0.2754 |
| IgA (g/L) (0/1/1)^#^ | 1.11 [0.84 ; 1.78] | 1.72 [1.38 ; 2.2] | 1.58 [1.04;2.15] | 0.2127 | 0.2961 | 0.5060 | 0.5060 |
| IgG (g/L) (0/1/1)^#^ | 7.46 [6.36 ; 8.65] | 9.68 [7.77 ; 11.04] | 9.85 [8.12;11.05] | 0.1151 | 0.2014 | 0.2014 | 0.6739 |
| IgM (g/L) (0/1/1)^#^ | 0.84 [0.63 ; 1.49] | 0.91 [0.72 ; 0.99] | 0.85 [0.64;1.08] | 0.8956 | 0.7385 | 0.7385 | 0.9055 |
| IL-1-beta (pg/ml) (1/2/2)^#^ | 35.41 [7.45 ; 98.97] | 55.4 [21.06 ; 72.34] | 59.63 [21.49;96.64] | 0.5655 | 1.0000 | 1.0000 | 1.0000 |
| IL-10 (pg/ml) (1/2/2)^#^ | 6.36 [1.82 ; 34.39] | 13.05 [4.12 ; 22.14] | 11.41 [2.95;23.33] | 0.7923 | 1.0000 | 1.0000 | 1.0000 |
| IL-17A (pg/ml) (1/2/2)^#^ | 20.19 [5.15 ; 102.81] | 34.75 [9.7 ; 56.68] | 34.22 [7.84;64.5] | 0.9492 | 1.0000 | 1.0000 | 1.0000 |
| IL-6 (pg/ml) (1/2/2)^#^ | 97.03 [27.04 ; 109.31] | 78.16 [56.02 ; 124.77] | 102.59 [57.52 ; 188.74] | 0.3928 | 0.9727 | 0.9727 | 0.9727 |
| IL-8 (pg/ml) (1/3/3)^#^ | 297.57 [39.23 ; 470.9] | 161.46 [62.64 ; 283.31] | 70.58 [9.13;440.59] | 0.3947 | 1.0000 | 1.0000 | 1.0000 |
| LAP (pg/ml) (1/2/2)^#^ | 14.86 [9.5 ; 20.42] | 14.36 [10.28 ; 19.05] | 13.95 [9.64;19.23] | 0.9781 | 1.0000 | 1.0000 | 1.0000 |
| TNF-alpha (pg/ml) (1/2/2)^#^ | 38.12 [18.09 ; 118.56] | 52.98 [29.68 ; 68.26] | 34.63 [23.27;70.31] | 0.7478 | 1.0000 | 1.0000 | 1.0000 |

Data are shown as median (interquartile range q1;q3).

^a^ The data is missing for 1 patient, the total number of HSP in these analysis is 59 (and not 60).

^#^ Missing data respectively for n patients in group HSP nephritis with anatomopathological documentation, HSP nephritis with urine test diagnosis, HSP without renal involvement.
