# Supplementary figures and images for: Regulatory T and B cells in pediatric Henoch–Schönlein purpura: friends or foes?
Source: Arthritis Res Ther. 2024 Feb 16;26:52. doi: 10.1186/s13075-024-03278-w (PMC10870453; doi:10.1186/s13075-024-03278-w)

## Slide 1
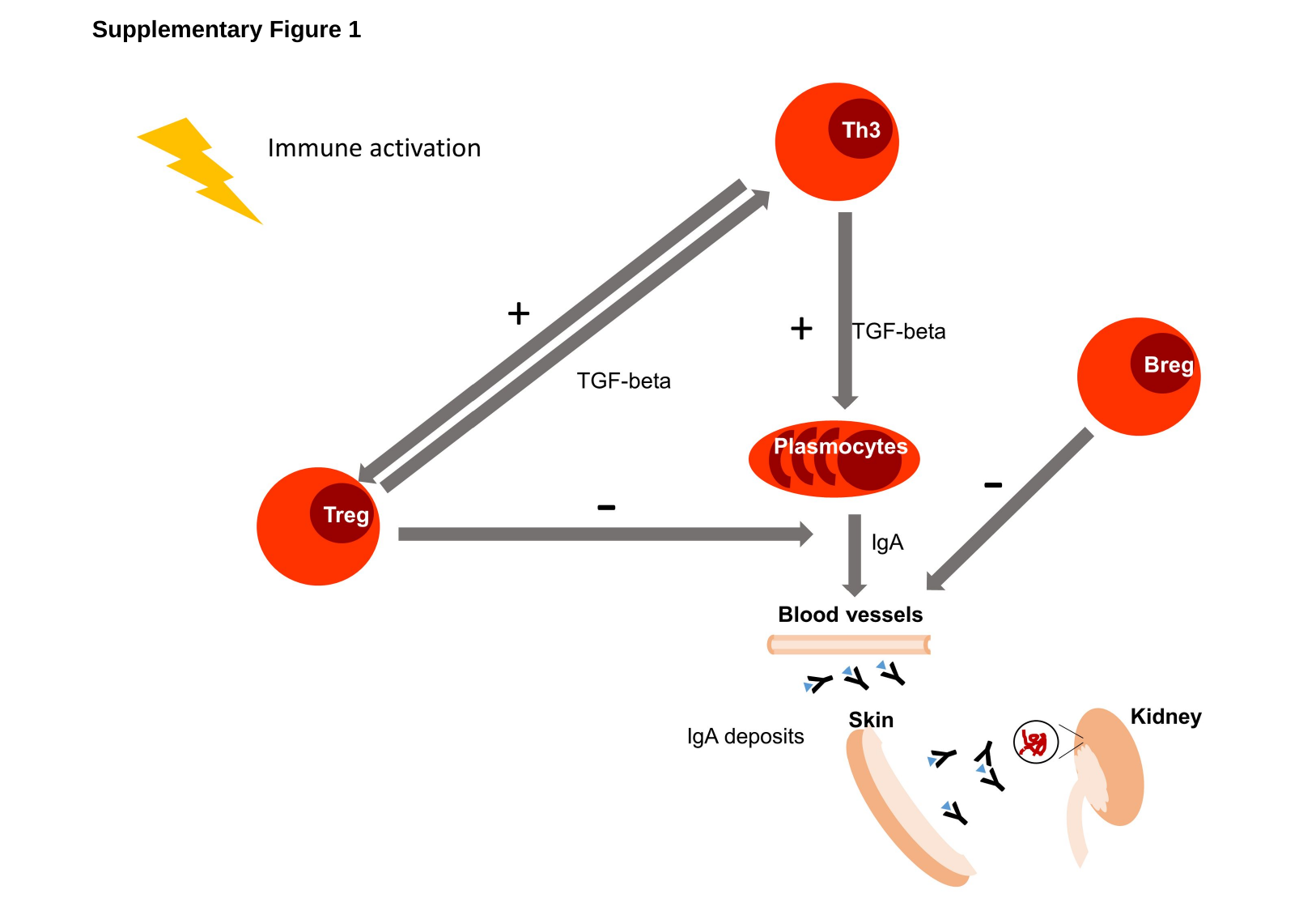

Supplementary Figure 1

Supplement: Supplementary file 3 — Additional file 3: Fig. S1. Model showing the role of regulatory B and T cells in pediatric HSP. [file 13075_2024_3278_MOESM3_ESM.pptx]
